# Effect of aging on the visuomotor control during continuous bimanual movement

**DOI:** 10.3389/fnagi.2025.1525535

**Published:** 2025-08-06

**Authors:** Kimia Kiani, Maya Patel, Qiushi Fu

**Affiliations:** ^1^Department of Mechanical and Aerospace Engineering, University of Central Florida, Orlando, FL, United States; ^2^Burnett School of Biomedical Sciences, University of Central Florida, Orlando, FL, United States; ^3^Biionix Cluster, University of Central Florida, Orlando, FL, United States

**Keywords:** bimanual coordination, motor adaptation, visual attention, visuomotor control, handedness, aging

## Abstract

**Introduction:**

Skilled bimanual coordination is an essential component of activities of daily living that relies on complex interactions between the limbs, yet how age-related changes impact asymmetries in visuomotor control during these tasks remains largely unknown. In the present study, we examined both motor performance and visual attention distribution in non-rhythmic continuous bimanual tasks and investigated the effect of aging.

**Methods:**

Twelve right-handed young adults (YA) and twelve right-handed older adults (OA) performed a bimanual tracking task in which each hand controlled a cursor using a robotic device to track the upward movement of a horizontal target line simultaneously and independently. We assessed participants’ performance in the symmetric condition, where both hands should perform the same actions to be successful. Additionally, participants performed the task in asymmetric conditions, where either a new force or a change in visuomotor gain was applied to only one hand, requiring participants to adapt by producing distinct actions with two hands. Overt visual attention was assessed by analyzing participants’ gaze fixation patterns during successful task performance.

**Results:**

Our findings revealed that YA experienced greater difficulty with asymmetric visuomotor constraints than asymmetric force constraints, whereas OA showed comparable performance challenges with both types of constraints. Moreover, we found that YA distributed the gaze consistently biased to the right side despite the effect of context asymmetry on tracking errors, while OA distributed their gaze more symmetrically. Lastly, YA demonstrated asymmetrical adaptation, with improved performance in the dominant right hand under left-sided constraints, while OA showed reduced adaptation capabilities.

**Discussion:**

These findings indicate that aging is associated with a reduction in lateralized attention and diminished adaptability to asymmetric task demands during bimanual visuomotor coordination.

## 1 Introduction

Skilled bimanual coordination is an essential component of a wide range of activities of daily living (Bailey et al., 2015; Kilbreath and Heard, 2005). Past investigations of the sensorimotor control of bimanual coordination have revealed complex interactions between two limbs at both behavioral and neural levels (Rueda-Delgado et al., 2014; Swinnen, 2002; Swinnen and Wenderoth, 2004). To maintain independent control of each limb, interhemispheric communication via the corpus callosum plays an important role during bimanual movements (Diedrichsen et al., 2003; Eliassen et al., 1999; Gooijers and Swinnen, 2014). Importantly, interhemispheric communication and the engagement of the two hemispheres during bimanual control may have a directional bias in relation to handedness, which is often defined as a preference in selecting which hand to perform unimanual tasks (Oldfield, 1971). It has been theorized that hemispheric lateralization could be underlying limb dominance such that dominant and non-dominant limbs have asymmetric sensorimotor capabilities to perform different types of unimanual tasks (Goble and Brown, 2008; Sainburg, 2014). Similarly, handedness and hemispheric lateralization could also be associated with asymmetric contributions to task performance during bimanual coordination (Mojtahedi et al., 2022; Serrien et al., 2006). For example, when two hands must produce in-phase rhythmic movement, the dominant hand tends to lead the non-dominant one (Swinnen et al., 1996). After mechanical perturbation was induced to bimanual rhythmic movement, phase adaptations were mostly made by the non-dominant left limb in right-handers (De Poel et al., 2008). Moreover, adding viscous load to the non-dominant left hand impaired the anti-phase rhythmic bimanual coordination more than adding load to the right hand (Serrien et al., 2003). These findings suggested that the dominant hemisphere may play a driving role in bimanual coordination, which has been supported by neural imaging studies (Pollok et al., 2007; Serrien, 2008; Walsh et al., 2008).

One explanation of the handedness-related directional bias in bimanual coordination could be an asymmetrical bias in allocating attention resources (Buckingham and Carey, 2015). It was found that the relative phase between limbs in rhythmic bimanual movement can be modulated by asking participants to direct visual attention to one limb (Amazeen et al., 1997; De Poel et al., 2008; Rogers et al., 1998; Treffner and Turvey, 1995). Similarly, shifting the focus of attention during bimanual reaching can alter the reaction times of subsequent unimanual reaching in a double-step reaching task (Buckingham and Carey, 2009). Together, these findings indicate that a rightward attention bias may exist during rhythmic and discrete bimanual tasks in right-handers. However, the relation between the visual attention bias and bimanual coordination was mostly examined in tasks with persistent contexts. To our knowledge, it has not been determined how handedness-related asymmetry in bimanual visuomotor control may adapt to the changes in task contexts that make bimanual coordination more difficult.

Furthermore, the effect of aging on the handedness-related asymmetry of bimanual visuomotor control also remains largely unknown since most research in this topic only examined young adults (YA). Previous research has shown that older adults (OA) often exhibit reduced ability to perform asymmetrical tasks, where independent control of each limb is necessary (Fling et al., 2011; Kiyama et al., 2014). Such age-related changes are thought to stem from diminished interhemispheric communication, which is crucial for coordinating dual-limb movements (Maes et al., 2017). Moreover, the natural decline in sensory feedback processing with age may further exacerbate difficulties in maintaining rhythm and phase alignment between limbs during complex bimanual tasks (Swinnen et al., 1998). Important, aging can also significantly alter how visual information is used in bimanual coordination. For instance, OA showed notable weaknesses in utilizing online visual control in bimanual reaching compared to YA (Coats and Wann, 2012), and an increased demand of attentional resources in challenging bimanual tasks (Lee et al., 2002). Additionally, in a task where active limb must follow the movement of the passively moved contralateral limb, directing central/foveal vision to the active limb impaired task performance in OA more than YA (Boisgontier et al., 2014). Nevertheless, these past studies of OA did not examine the distribution of visual attention nor the adaptation to changing task contexts.

In the present study, we quantified both motor performance and gaze distribution in a free-viewing bimanual non-rhythmic tracking task using a pair of robotic manipulanda with both OA and YA. This experimental design enabled us to determine the effects of aging on bimanual visuomotor control when symmetric bimanual actions must be performed continuously with high precision, as well as under conditions of asymmetry introduced by applying unilateral constraints, either a new force or a visuomotor gain change. Moreover, we can determine the asymmetry of the overt visual attention with the measurement of the gaze distribution in these experimental tasks. We hypothesized that YA perform better with their dominant limbs and direct their gazes more frequently to the dominant side. Additionally, we hypothesized that the visuomotor behavior of OA would be less asymmetrical between two limbs than those of YA.

## 2 Materials and methods

### 2.1 Participants

This study enrolled twenty-four healthy individuals, ensuring that none had suffered neuromuscular injuries to their upper bodies and had normal to good vision. The participants were divided equally into two age groups. The YA group consisted of twelve participants (5 females, age 21 ± 1.2 years, mean ± S.D.), and the OA group consisted of twelve participants (9 females, age 63 ± 5.4 years). Using a modified version of the Edinburgh handedness inventory, we determined the laterality index (LI, a score between −100 and 100) for each participant. All participants were right-handed (YA LI and OA LI were 82.9 ± 13.56 and 81.6 ± 19.34, respectively). All participants performed the same experimental protocol described below, which was approved by the Institutional Review Board at the University of Central Florida according to the Declaration of Helsinki (STUDY00001351). All participants gave informed consent to participate in this study.

### 2.2 Protocol

**FIGURE 1:**
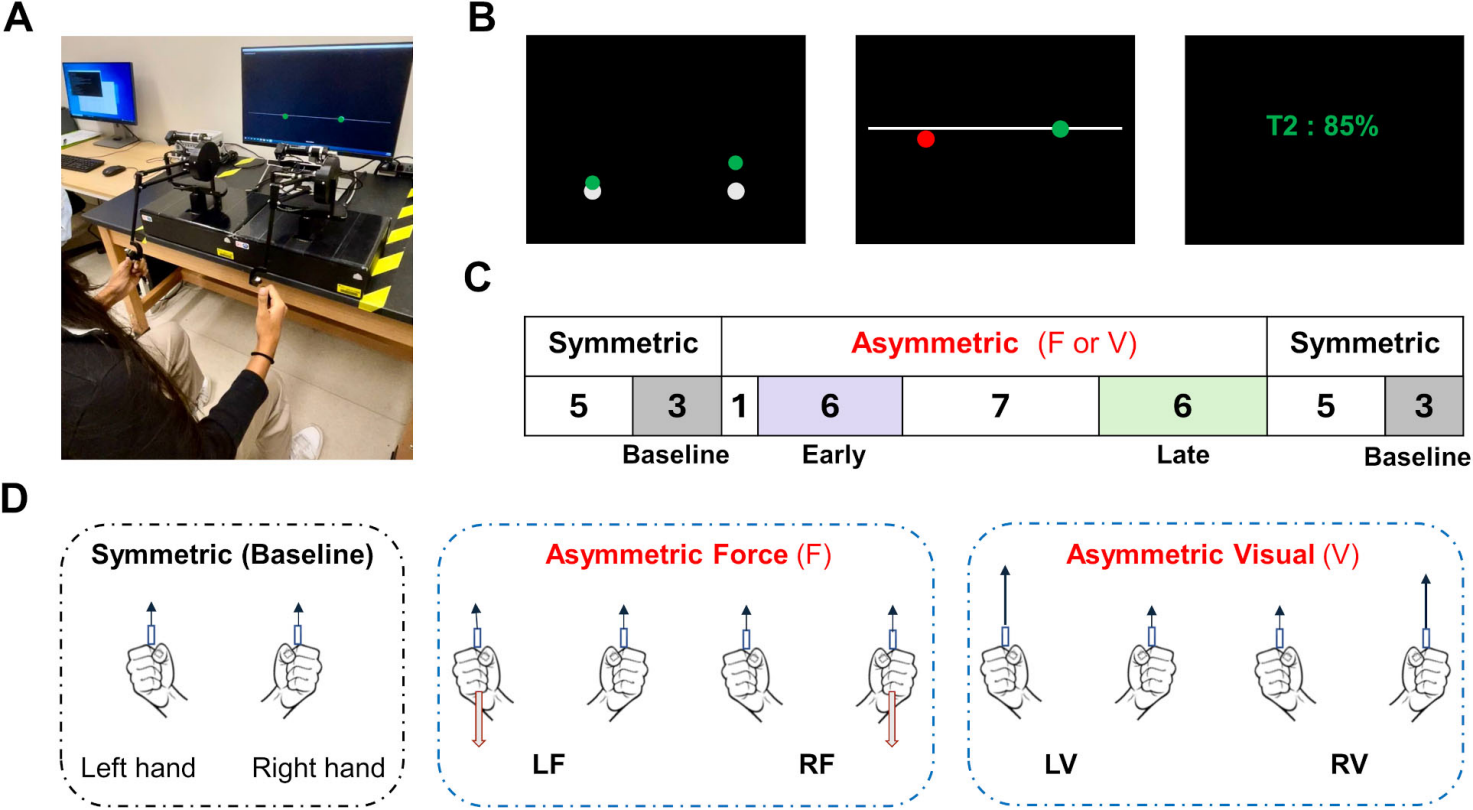
Bimanual tracking task setup and conditions. **(A)** Experimental setup; Participants operate the end effector of each robot, controlling the corresponding cursors on the screen. **(B)** Visual display; Depicts three task states within a trial, starting with two white cursors as start regions. A green circle indicates the right hand is successfully tracking a moving bar, while a red circle signals the left hand’s failure to maintain an appropriate distance. A green percentage displayed on the screen quantifies the score based on the proportion of time both cursors were green. **(C)** Trial sequence: each block consists of 36 trials. The numbers in the table represent the number of trials, with shaded areas highlighting those included in data analysis. **(D)** Task conditions; Outlines Baseline symmetric conditions and asymmetric conditions, either with added force (F) or altered visuomotor sensitivity (V), which demand different motor commands for each hand.

This study used a bimanual tracking task which was implemented with two Phantom 1.5HF robots (3D Systems, Rock Hill, SC) located on the table in front of a computer monitor (Figure 1A). Participants were seated on a chair without armrests with their elbows flexed at an approximate 90-degree position. The distance between the monitor and the participant’s eyes is approximately 1 m. Participants were told to hold the handle firmly and that moving the handles would translate to movements of two cursors (1 cm diameter) displayed on the screen. The visual angle between the two cursors is approximately 20 degrees. Only the upward movements of the robots are mapped to the upward movement of the cursors, which was recorded at 500 Hz. We implemented a soft virtual spring-damper in the anterior-posterior direction (spring constant, Kw = 0.25 N/mm; damping constant, Bw = 0.03 N⋅s/mm) for each robot, such that the participants’ movements were constrained to a vertical plane (x-y) that was aligned with their frontal plane. The constraints would generate force to gently push the hand back to the plane if the participants’ hands deviate from the plane. These robots are lightweight and highly back-drivable, thus requiring minimal effort to move within the constraint plane. The experimental task was implemented with the CHAI3d software library (Conti et al., 2005) to control the robots and render visual feedback. Participants were instructed to find a comfortable horizontal distance between the hands to perform the task, although the horizontal movements of their hands were not mapped to the cursors.

Before each trial, a white sphere (1.5 cm diameter) was located at the bottom of the screen for each cursor (Figure 1B, Left). Participants were instructed to initiate a trial when they are ready by moving the cursors into the white spheres. A trial began after the centers of the cursors were placed inside the white spheres for more than 1 s. As the trial started, the white spheres disappeared and a horizontal white line that spans throughout the screen began to move upward at the speed of 1.4 cm/sec. Participants were instructed to track the moving line with the cursors, by placing the cursors on the moving line. When the distance between the center of each cursor and the line was less than 5 mm, the cursor turned green. Tracking was considered successful when both cursors were green simultaneously. Otherwise, if either cursor failed to track the line, its color immediately turned red (Figure 1B, Middle). Each trial was 7 s long and at the end of each trial, a percentage score was displayed on the screen, indicating the proportion of time during which both cursors were green. Participants were encouraged to attain a high score (Figure 1B, Right).

There were four blocks of 36 trials (Figure 1C). Participants performed blocks in random order to minimize the effect of order in the proposed task. Each block included 16 trials in a symmetric context (Trials 1–8 and 29–36) in which the movement mappings between the robots and cursors were the same (Figure 1D) and 1 cm robot movement translates to 1 cm cursor movement. These trials were designed to define the Baseline performance of the bimanual tracking task, as well as to wash out the motor adaptation from other preceding task contexts. The middle trials of each block (Trials 9–29) were in asymmetric task contexts where a constraint was applied to one of the limbs. There were two types of task constraints: Force (F) and Visuomotor (V). In F conditions, the robot/cursor movement mapping remains the same but a constant load of 4 N was applied by the robot in the downward direction to the target limb (Figure 1D). In contrast, the V conditions implemented a change in the robot/cursor movement mapping such that the target limb controls the cursor with a higher sensitivity, i.e., 1 cm robot movement translates to 2 cm cursor movement (100% increase from the symmetric condition, Figure 1D). Note that both the F and V conditions can make the tracking of the target limb more difficult. The kinetic load required the limb to move the load with a higher level of muscle contraction which would increase the signal-dependent noise of the motor system (Jones et al., 2002), whereas high visuomotor sensitivity would magnify the movement error of the limb. Additionally, these asymmetric task contexts required two limbs to produce asymmetric motor commands, which may be subject to inter-limb coupling effects. All participants performed five familiarization trials with the symmetrical condition before performing all blocks of trials of the task.

### 2.3 Data analysis

#### 2.3.1 Behavioral performance

We developed a custom program in MATLAB (MathWorks, Natick, MA, USA) to quantify the performance of the participants and their visual attention allocation over different contexts of the experiment. We defined three trial stages of Baseline, Early and Late stage in which we can quantify visuomotor behavior across a few trials within the same task conditions to reduce the effect of trial-to-trial variability (Figure 1C). The Baseline stage comprises the last three trials in a symmetric context before and after the trials in asymmetric contexts, i.e., Trials 6–8 and 34–36. All Baseline trials from the four blocks are pooled together (24 trials total). The Early stage of each asymmetric condition consists of the six trials after the first trial in asymmetric contexts (Trials 10–15), whereas the Late stage includes the final six trials in asymmetric contexts (Trials 23–28). We did not include the first trial of the asymmetric contexts in the early stage to reduce the potential confound of surprise, although participants were aware of the trials’ sequence and expected a consistent constraint to be applied starting at Trial 9 and ending at Trial 29. Note that Early and Late stages were not pooled across different asymmetric contexts. Within these three trial stages, we computed the score and tracking Error for each hand as the key behavioral variables to assess motor performance in our tasks across different task contexts. A score was displayed at the end of each trial, which was calculated as the ratio between the time during which both limbs were successfully tracking the moving bar and the total duration of the trial (in %). We averaged the scores across all trials within each stage. This variable quantifies participants’ overall task performance in each trial stage. Additionally, we defined the tracking Error for each arm as the ratio between the duration of time during which the arm failed to track the moving bar to the total duration of all trials within each trial stage. Note tracking errors can occur simultaneously for both arms or only for one side, both of which would cause a lower performance score.

#### 2.3.2 Gaze data

Gaze was found to lead hand movement in tasks that involve a sequence of precise motor actions (Johansson et al., 2001), which highlights the importance of fovea-anchored coordinates in predictive hand movement planning and monitoring critical events for verification of task progress. This study used a GP3 eye tracker system (Gazepoint, Vancouver, BC, Canada) to record the participants’ gaze across the screen. The eye tracker was calibrated with a 9-point calibration for each participant and the sampling rate was 60 Hz. One way to quantify the distribution of visual attention was to determine how frequently the gaze was directed to one side of the task. We first linearly interpreted the gaze location data to fill the gaps where the gaze tracking was not available (e.g., blink). Then we identified non-saccade gaze segments where the velocity of the gaze movement on the screen was below 35 degree/s (Wass et al., 2013). Also, gaze segments that were shorter than 100 ms were discarded because they can be considered as short saccades and maybe beyond the limits of accuracy of our eye-tracker (Wass et al., 2013). To minimize the inclusion of gaze fixations that were associated with error correction, we excluded gaze fixation segments that started with one or two sides making tracking Errors. During these moments, the cursor changed color to indicate an error, likely drawing the participant’s gaze toward the side of the disruption. Therefore, gaze fixation segments that started with one or two sides making tracking Errors were excluded to ensure that our analysis focused on overt attention in neutral, task-driven conditions, uncontaminated by visually triggered error responses. Note that our task was dynamic and involved constantly slow-moving targets, so these data segments can represent either fixation (fixating on a stationary point) or smooth pursuit (fixating on a moving object). However, we did not distinguish between these two types in our analyses and defined them as general “gaze fixations.”

For each included gaze fixation segment, we computed the average horizontal coordinates and labeled the segment as a Left one or a Right one separated by the mid-line of the screen. The **Fixation Frequency Asymmetry** was defined as the difference between the number of gaze fixations that were labeled as Left and Right, averaged within each trial stage. A positive and a negative value would indicate gaze fixations were more frequently directed toward the right side and left side, respectively. An alternative way of quantifying visual attention is to determine how much time a gaze fixation may last. The average duration of gaze fixations was calculated using the sum of the durations of all fixations toward a specified side (either left or right) within a trial stage, divided by the total number of fixations toward that side. The **Fixation Duration Asymmetry** was then defined as the difference between the average duration of fixations for the right side and left side. A positive and a negative value of the Fixation Duration Asymmetry metric indicated a single gaze fixation tends to last longer when it’s on the right side and left side, respectively. Both above-mentioned metrics quantify the asymmetry of the gaze distribution, which can be interpreted as the extent to which overt visual attention may be biased to the right or left side.

### 2.4 Statistical analysis

All statistical analyses were performed in SPSS software (IBM, Armonk, NY, USA). We first directly compared between the two Age groups for the Baseline symmetric condition using two-samples *t*-tests. These tests were implemented for score, Fixation Duration Asymmetry, Fixation Frequency Asymmetry and tracking Error. Given the differences between two age groups uncovered in the Baseline conditions, we decided to only perform within-group repeated-measure ANOVA for the asymmetric conditions where adaptation occurred. Therefore, OA and YA were analyzed separately following the same statistical design as following. During each asymmetric block, a specific type of constraint (force or visual, F or V) was applied to one side (left or right). This resulted in eight distinct asymmetric conditions based on the constraint type, side, and trial stage (LV Early, LV Late, RV Early, RV Late, LF Early, LF Late, RF Early, RF Late). We used three-way repeated ANOVA to investigate the effect of (constraint) Type, (constraint) Side, and (trial) Stage on score, Fixation Duration Asymmetry, and Fixation Frequency Asymmetry. Significant interactions were then examined with *post hoc* comparisons with Bonferroni corrections. Additionally, we also used paired *t*-tests to compare each asymmetric condition to the symmetric Baseline condition.

For the tracking Error from each hand, we separated the analysis by constraint side because we found that the adaptation process and gaze behavior may be different after constraint was introduced to the left and right sides. Furthermore, we also computed ΔError to quantify the change of Error in asymmetric contexts from the corresponding Baseline value. For each constraint side, we analyzed ΔError using three-way mixed ANOVAs with three factors of (constrained) Hand, (constraint) Type, and (trial) Stage. Significant interactions were then examined with *post hoc* comparisons with Bonferroni corrections. Lastly, we also used one-sample *t*-tests to examine whether ΔError differed from zero, which would indicate a change from the symmetric Baseline context.

## 3 Results

### 3.1 Task performance

#### 3.1.1 Score in symmetric condition for YA and OA

We first compared the score from symmetric trials 6–8 to trials 34–36 within each age group using paired-sample *t*-tests and found no difference. This justified the pooling of these trials together as the Baseline condition. During the Baseline condition, YA participants obtained scores of 68.63 ± 7.57% (we report mean ± S.D. in the main text). In contrast, OA’s average score in the symmetric Baseline condition was worse than YA (51.99 ± 19.21%), suggesting that accurate tracking of both hands was achieved only half of the time. Our statistical analysis confirmed this observation that the performance of the YA was significantly higher than OA (*t*-test, *p* = 0.01).

#### 3.1.2 Score in asymmetric conditions for young adults

**FIGURE 2:**
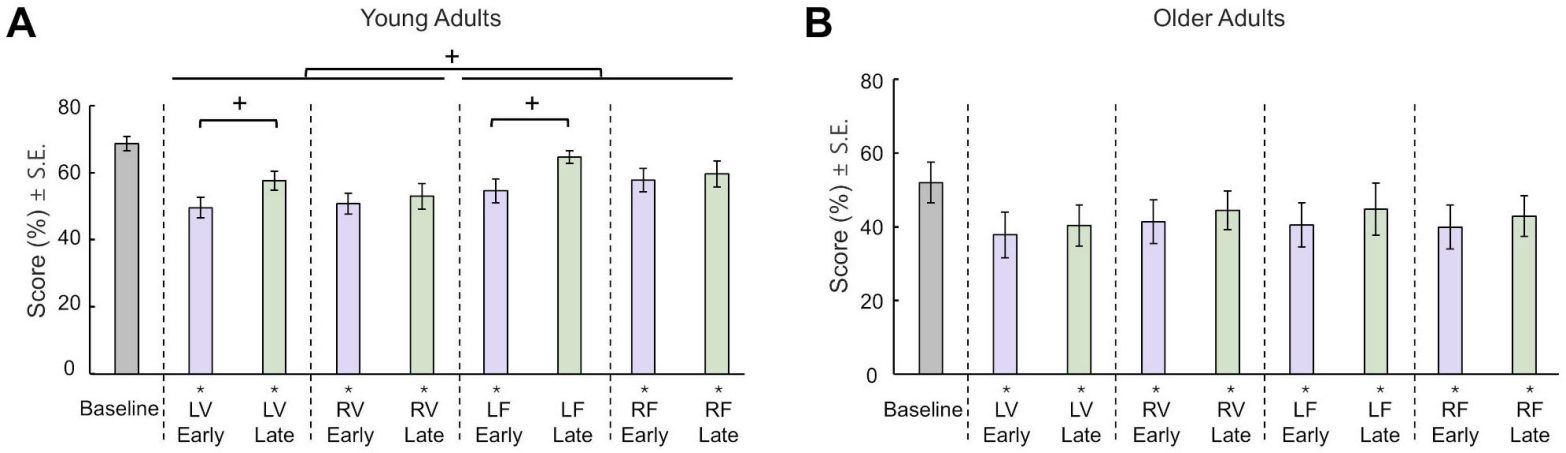
Task performance (mean ± S.E.). **(A)** Average score of YA for different blocks. **(B)** Average score of OA for different blocks. Asterisks denote significant differences from Baseline and the crosses denote significant differences between stages.

We observed an initial decrease in performance resulting from applied constraints which revealed that adding load to one limb (F conditions) had less impact on the performance than changing the visuomotor sensitivity of the limb (V conditions). Moreover, it was easier for the participants to adapt to the changes that were applied to the left (non-dominant) side (Figure 2A). These observations were confirmed by a three-way mixed ANOVA (Type × Side × Stage) showing significant effects of Type (F (1, 11) = 8.33, *p* = 0.015), as well as significant Side × Stage interaction (F (1, 11) = 9.352, *p* = 0.011). *Post hoc* comparisons revealed that YA performance significantly improved from Early to Late stages in LV and LF conditions (*p* = 0.007 and *p* = 0.002, respectively), but not in RV and RF conditions (*p* > 0.38). Overall, these results suggest that, after adding constraints on one limb during bimanual movements, YA’s ability to adapt to the new task that requires asymmetric limb actions may depend on which limb is subjected to the constraint. Furthermore, one-sample *t*-tests showed that the YA score was significantly different in asymmetric blocks compared to the Baseline (*p* < 0.015) except in the late trial stage of the LF context.

#### 3.1.3 Score in asymmetric conditions for older adults

In the symmetric Baseline condition, OA’s average score was 51.99 ± 19.21%, suggesting that accurate tracking of both hands was achieved approximately half of the time. The overall score for the constrained trials were lower than the Baseline. It was observed that imposing a load on one limb (F conditions) had a similar impact on performance as altering the visuomotor sensitivity of the limb (V conditions). Furthermore, OA did not exhibit adaptation to the induced constraints throughout the stages of the experiment. These observations were confirmed by a three-way mixed ANOVA (Type × Side × Stage) showing no significant main effects or interactions of score. Furthermore, one-sample *t*-tests showed that score were significantly lower than zero (*p* < 0.016) in all asymmetric conditions (Figure 2B).

### 3.2 Visual attention

#### 3.2.1 Gaze distribution in symmetric condition for OA and YA

**FIGURE 3:**
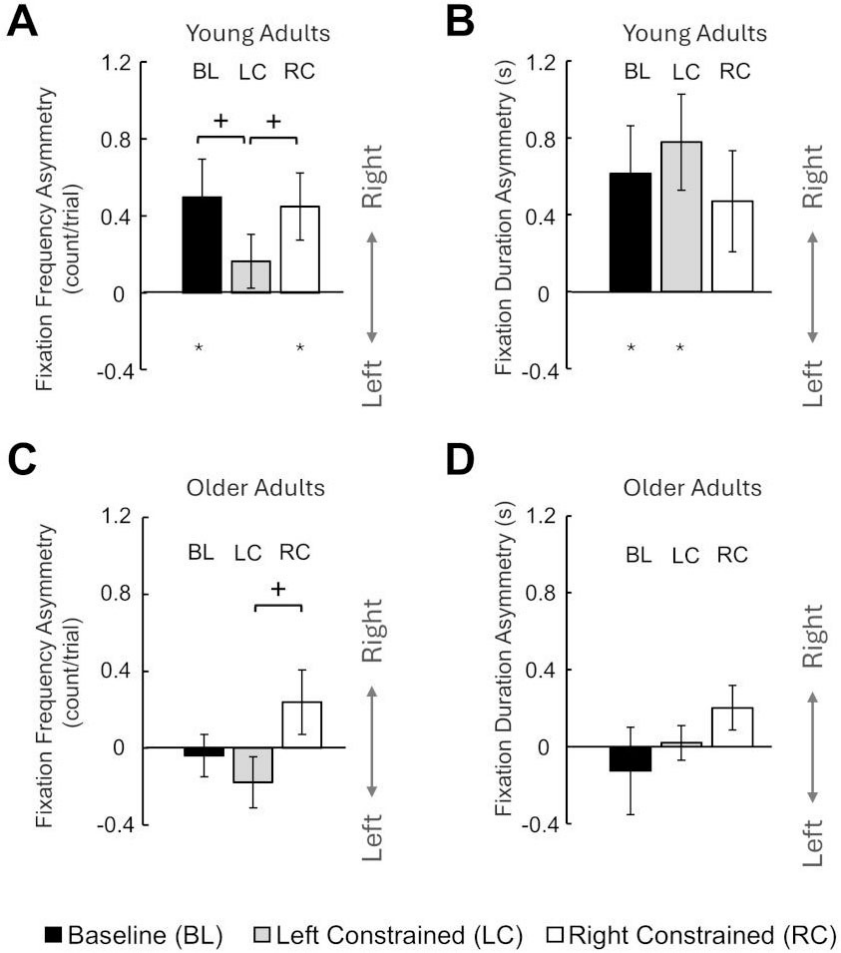
Gaze fixation in symmetric (Baseline) and asymmetric contexts with left- and right-side constraints (mean ± S.E.). **(A)** Fixation Frequency Asymmetry for YA. **(B)** Fixation Duration Asymmetry for YA. **(C)** Fixation Frequency Asymmetry for OA. **(D)** Fixation Duration Asymmetry for OA. Asterisks denote significant differences from zero (symmetric), and crosses denote significant differences between conditions.

To quantify the distribution of visual attention we defined gaze fixation frequency which demonstrates on average how often the gaze was directed to one side of the screen during each stage. The gaze fixation of YA during the Baseline revealed a rightward asymmetry of gaze, and participants tended to allocate their attention toward the right side (3.60 ± 1.57 counts/trial) more than the left side (3.10 ± 1.47 counts/trial). One sample *t*-test confirmed these gaze observations and showed that Fixation Frequency Asymmetry during Baseline was significantly greater than zero (*p* = 0.029; Figure 3A). In contrast, OA’s results showed that during the Baseline, the frequency at which participants allocated their gaze toward the right and left side was symmetrical (2.37 ± 1.06 counts/trial, and 2.41 ± 1.22 counts/trial, respectively). A one-sample *t*-test confirmed that Fixation Frequency Asymmetry during Baseline was not significantly different from zero (Figure 3C). Additionally, *t*-test revealed that the Fixation Frequency Asymmetry during the Baseline condition was significantly greater in YA compared to OA (*p* = 0.027).

We also calculated the Fixation Duration Asymmetry to determine in which side the gaze fixation tends to last longer. During the Baseline symmetric condition, YA allocated their attention for a longer duration to the right side (1.07 ± 0.90 s) compared to the left side (0.45 ± 0.29 s). A one-sample *t*-test confirmed these findings and showed that Fixation Duration Asymmetry during Baseline was significantly greater than zero (*p* = 0.031; Figure 3B). In contrast, OA showed similar fixation duration in the right and left side (0.76 ± 0.12 s and 0.89 ± 0.23 s, respectively). One-sample *t*-test confirmed this observation and showed Fixation Duration Asymmetry of OA during Baseline was not significantly different from zero (Figure 3D). Additionally, *t*-test showed that the Fixation Duration Asymmetry was significantly greater in YA compared to OA during symmetric condition (*p* = 0.038). Overall, these results suggest that YA had an asymmetric distribution of overt visual attention in the symmetric conditions, but OA did not.

#### 3.2.2 Gaze distribution during asymmetric conditions in young adults

A three-way ANOVA (Type × Side × Stage) of Fixation Frequency Asymmetry showed no significant effect of Type or Stage. However, the side to which the constraint has been applied influenced the gaze fixation. We observed more gaze fixation for those blocks with constrained applying to the right side compared to the left side. Three-way ANOVA (Type × Side × Stage) confirmed this by showing significant effects of Side (F (1, 11) = 5.012, *p* = 0.047). Therefore, we used the average value of Fixation Frequency Asymmetry separately computed for conditions with right-side constraints (0.45 ± 0.63 counts/trial) and left-side constraints (0.16 ± 0.35 counts/trial). Additionally, one-sample *t*-tests showed that Fixation Frequency Asymmetry during both right- and left-side constrained conditions was also significantly greater than zero (*p* = 0.029). Lastly, paired *t*-tests revealed that the Fixation Frequency Asymmetry in left, but not right, constrained conditions was significantly smaller than that in the symmetric Baseline condition (*p* = 0.014).

Moreover, three-way ANOVA (Type × Side × Stage) of Fixation Duration Asymmetry showed no significant main effect and no interaction. Consistent with our analysis for OA, we calculated the average Fixation Duration Asymmetry separately for the right- and left-side constrained conditions. Our results indicated that the average Fixation Duration Asymmetry in the left-side constrained conditions (0.78 ± 0.65 s) was greater than zero (one-sample *t*-test, *p* = 0.010), but not in right-side constrained conditions (0.47 ± 0.65 s). Lastly, paired *t*-test revealed that the Fixation Duration Asymmetry in asymmetric conditions was not statistically different from the Fixation Duration Asymmetry in the symmetric Baseline condition (Figure 3B). Overall, the YA consistently exhibited an asymmetrical gaze distribution favoring the right side across various experimental conditions and stages.

#### 3.2.3 Gaze distribution during asymmetric conditions in older adults

We observed that, after the asymmetric constraints were applied, the gaze distributions were not systematically different between visual or force asymmetric conditions. However, the side to which the constraint has been applied may influence the gaze fixation. Our observation revealed more gaze fixation for those blocks with constrained applying to the right side compared to the left side Three-way ANOVA (Type × Side × Stage) confirmed this by showing significant effects of Side (F (1, 11) = 12.022, *p* = 0.005). Therefore, we averaged the Fixation Frequency Asymmetry value across early and late stages separately for right-side and left-side constrained conditions (0.24 ± 0.58 counts/trial, −0.18 ± 0.46 counts/trial). One-sample *t*-test showed that Fixation Frequency Asymmetry was not significantly different from zero in these asymmetric conditions.

Also, further gaze analysis revealed that the changes of Fixation Duration Asymmetry from Baseline values during constrained conditions were not systematically different across conditions. A three-way ANOVA (Type × Side × Stage) did not show significant main effects or interactions. However, consistent with our Fixation Frequency Asymmetry analysis, we averaged the value of Fixation Duration Asymmetry separately for left- and right-side constraints across all stages (0.02 ± 0.30 s, 0.20 ± 0.28 s). One-sample *t*-test showed that the Fixation Duration Asymmetry was not significantly different from zero in these asymmetric conditions. Additionally, the *t*-test revealed that the Fixation Duration Asymmetry in asymmetric conditions was not statistically different from the Fixation Duration Asymmetry in the symmetric Baseline condition.

### 3.3 Error

#### 3.3.1 Tracking errors in the left and right hands during symmetric conditions

**FIGURE 4:**
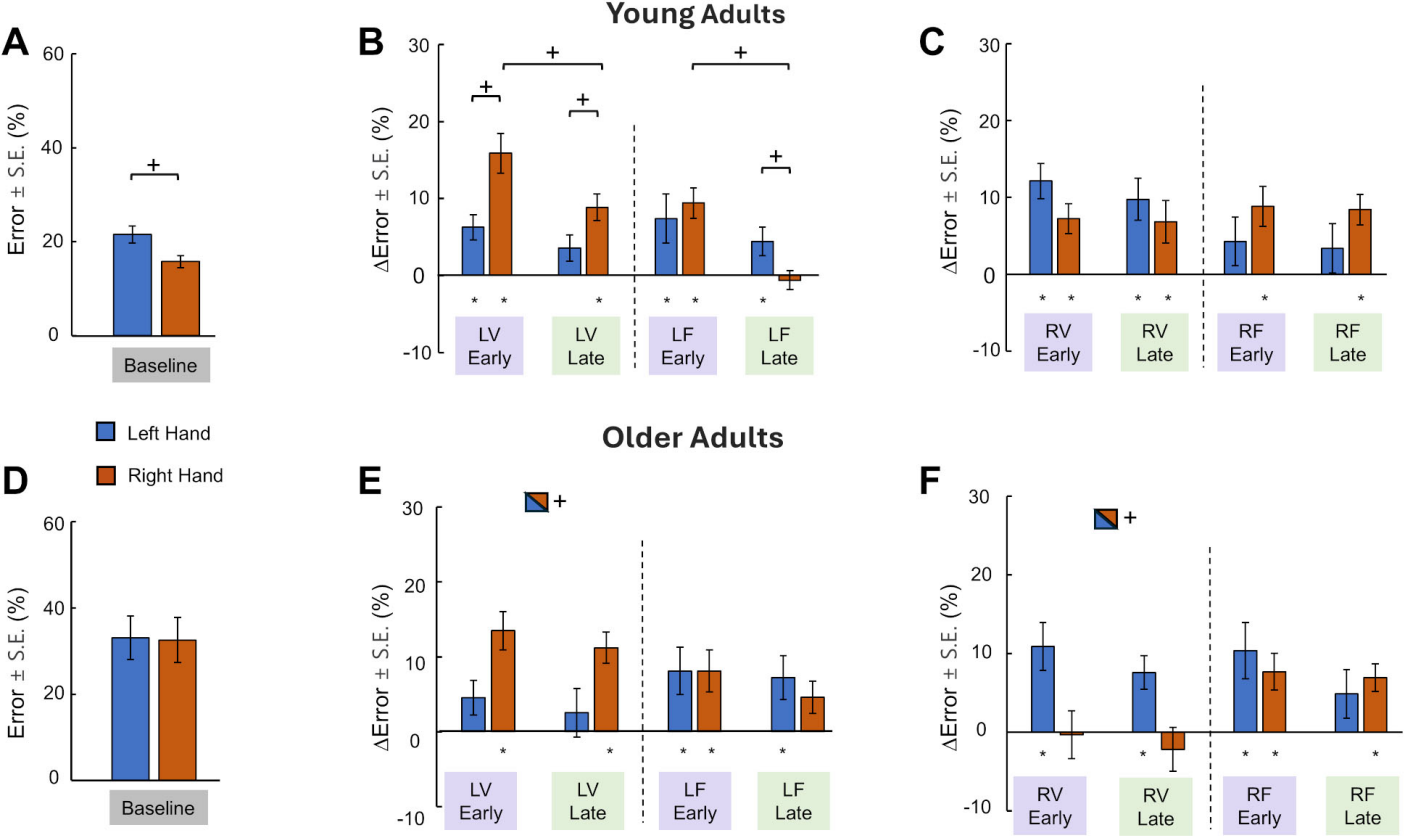
Error made by left and Right hand (mean ± S.E.). **(A)** Young adults’ Error in symmetric Baseline context. **(B)** Young adults’ ΔError asymmetric contexts with left side constraints. **(C)** Young adults’ ΔError asymmetric contexts with right side constraints. **(D)** Older Adults’ Error in symmetric Baseline context. **(E)** Older Adults’ ΔError for asymmetric contexts with left-side constraints. **(F)** Older Adults’ ΔError for asymmetric contexts with right-side constraints. Asterisks denote significant differences from baseline, and crosses denote significant differences between conditions.

Young adults showed different performances of each limb during the symmetric Baseline context. Paired *t*-test revealed that the tracking Error of the right hand was smaller compared to the left hand (*p* = 0.002; Figure 4A). However, OA’s result showed that the tracking Error was statistically the same for the right (32.6 ± 18.1%) and the left hand (33.1 ± 17.5%) during the Baseline (Figure 4D). To compare YA and OA directly in the Baseline condition, we calculated the difference in the tracking error between the right hand and left hand separately for each age group. A *t*-test showed that the tracking error difference was significantly larger in YA than in OA (*p* = 0.047). Overall, these results suggest that the right hand only had an accuracy advantage over the left hand in YA but not OA for the symmetric context.

#### 3.3.2 Tracking errors of the left and right hands during asymmetric conditions for young adults

To investigate the extent to which tracking Error changed for each hand in asymmetric contexts, we examined the conditions with left-side and right-side constraints separately. After the introduction of constraints to the left side (either LV or LF), it was observed that tracking Error in both hands increased. After repeated exposure to these unilateral constraints, the tracking Error was only reduced in the right but not the left hand (Figure 4B). Such dependency on hand dominance was confirmed by ANOVA showing significant Hand × Stage interaction (F (1, 11) = 8.04, *p* = 0.016), and Hand × Type interaction (F (1, 11) = 5.786, *p* = 0.035). *Post hoc* comparisons revealed that the ΔError of the right hand was significantly larger than that of the left hand in the Early trial stage only for the LV condition (*p* = 0.016), but not for the LF condition. In the Late trial stage, the ΔError of the right hand was still significantly larger than that of the left hand for the LV condition (*p* = 0.025), but smaller than that of the left hand for the LF condition (*p* = 0.036). Additionally, only the right hand showed a significant reduction of ΔError from the Early to Late stages (*p* = 0.02 for LV, *p* < 0.01 for LF). Lastly, one-sample *t*-tests showed that ΔError was significantly larger than zero in LV Early, LV Late, and LF Early for the right hand, as well as LV Early and LF Early and Late for the left hand (Figure 4B). These results suggest that the overall performance improvement in the asymmetric contexts with left-side constraints was primarily driven by the adaptation of the right hand, whereas the adaptation of the left hand was minimal. Furthermore, visuomotor and force constraints affected right hand tracking differently, whereas the Error increase in the left hand was not dependent on the type of constraint.

After the constraints were applied to the right side, there was no reduction of tracking Error across trial stages on either hand (Figure 4C). We also observed that the effect of constraint type on tracking Error differed between L and V conditions. This was confirmed with ANOVA showing a significant Hand × Type interaction (F (1, 11) = 5.595, *p* = 0.037). However, *post-hoc* tests did not find significance in pair-wise comparisons. Additionally, one-sample *t*-tests revealed that ΔError was significantly greater than zero for both hands in all RV conditions (*p* < 0.01), whereas only the right hand but not the left hand had non-zero ΔError (*p* < 0.01). Overall, the YA’s results suggest that the left hand responded to rightward constraints differently based on the type of constraints such that the tracking Error increased in the visuomotor condition, but not in the force condition. In contrast, whereas right hand increased tracking Error in both constraint types.

#### 3.3.3 Tracking errors of the left and right hands during asymmetric conditions for older adults

For OA, consistent with the YA analysis, we examined the conditions with left-side and right-side constraints separately. During the asymmetric condition, when constraints were imposed on the left side (either LV or LF), the responses of each hand were dependent on the type of the constraints. Specifically, the visual constraint led to more increased Error in the right hand than the left hand, whereas the force constraint did not result in different Error increases between the hands (Figure 4E). This observation was confirmed by a three-way ANOVA (Hand × Type × Stage) showing a Hand x Type interaction (F (1, 11) = 10.34, *p* = 0.008). *Post hoc* comparisons revealed that the ΔError of the right hand was significantly larger than that of the left only for the visual asymmetric condition (*p* = 0.006), but not for the force condition. Also, the ΔError for the right hand was significantly larger for the visual asymmetric condition applied to the left side (*p* = 0.016) compared to the ΔError for force asymmetry Lastly, one-sample *t*-tests showed that ΔError was significantly larger than zero in LV Early, LV Late, and LF Early for the right hand, as well as LF Early and Late for the left hand during the left-side asymmetric condition (Figure 4E).

After the constraints were applied to the right side, changes in the Error for each hand were again different in response to either visual or force asymmetry. Interestingly, when the visual constraint was imposed on the right hand, the tracking accuracy was not changed compared to their performance during the symmetric context (Figure 4F). However, the Error in the left hand increased from the Baseline for both types of rightward asymmetry (RV and RF). This observation was confirmed by a three-way ANOVA (Hand × Type × Stage) showing a Hand x Type interaction (F (1, 11) = 5.76, *p* = 0.035). *Post hoc* comparisons revealed that the ΔError of the Left hand was significantly larger than that of the right hand only for the visual asymmetric condition (*p* = 0.015), but not for the force condition. Also, the ΔError for the right hand was significantly larger for the visual asymmetric condition applied to the right side (*p* = 0.013) compared to the ΔError for force asymmetry. Lastly, one-sample *t*-tests showed that ΔError was significantly larger than zero in RF Early and Late for the right hand, as well as RF Early, RV Early, and Late for the left hand. Overall, these OA results suggest that the visual constraint caused the tracking accuracy of the contralateral side to decrease, but the force constraint increased tracking Error for both hands.

## 4 Discussion

### 4.1 Asymmetrical visuomotor constraint was more difficult than asymmetrical force constraints in YA, but not OA

Extensive research has demonstrated that bimanual coordination is more difficult to perform in asymmetric contexts than symmetric ones in a broad range of tasks. Movement errors were greater when two limbs must produce asymmetric trajectory than symmetric ones (Buchanan and Ryu, 2012; Kirby et al., 2019; Wenderoth et al., 2006). Our results with visuomotor asymmetry were consistent with these studies. Regarding force constraints, Serrien et al. (2003) showed that adding load to rhythmic wrist movement caused deterioration of anti-phase bimanual coordination. Additionally, it has been shown that error was greater when two hands must produce asymmetric amplitudes than symmetric ones in an isometric force production task (Kennedy et al., 2017). Our results with load asymmetry were also consistent with these studies. Lastly, OA in our study performed worse than YA in both symmetric and asymmetric contexts, and the difficulty of performing asymmetric bimanual tasks persisted in OA. This is in general agreement with the broad literature that shows age-related deficits in bimanual coordination (Kang et al., 2022).

Importantly, our results extend from previous research by using a unique task that requires continuous, non-rhythmic movement from two limbs. Most bimanual studies used tasks that require either rhythmic movement that follows external timing cues or short-duration discrete movements. It has been theorized that both rhythmic and discrete movement are different types of building blocks, i.e., motor primitives, of more complex motor behaviors (Hogan and Sternad, 2012; Howard et al., 2011; Wiegel et al., 2020). Because our tasks require relatively slow movement, it is more likely to be controlled by combining a series of discrete primitives with emergent timing, i.e., submovements (Park et al., 2017). Therefore, our results, combined with previous studies, indicate that the inter-limb coupling is independent of the temporal organization of movements in both YA and OA.

Visuomotor constraints and load constraints have both been used extensively in previous bimanual research. However, a direct comparison between the effect of these two types of constraints was difficult because both the main bimanual task and constraint implementation varied substantially across studies. Nevertheless, a recent study using bimanual reaching showed that unilateral adaptation to visuomotor rotation of the right hand caused more interference on the invisible left hand than the adaptation to dynamic perturbation (Desrochers et al., 2021). This is consistent with our results that demonstrated a larger performance error with visuomotor constraints than load constraints across both early and late adaptation stages in YA (Figure 2B). Specifically, the visuomotor constraints in our study required two limbs to produce asymmetrical movement kinematics with different end-effector and joint space amplitudes. In contrast, the load constraints we used did not alter the spatial aspects of the movement but only required asymmetrical muscle contraction levels. The control of upper limb movement is a process that involves the transformation of coordinate frames from high-level spatial representations to low-level muscle activations across a large frontoparietal network (Filimon, 2010). It has been shown that these different coordinate systems may follow a cortical distribution gradient where spatial representations are more likely to be encoded in the parietal cortex, the joint and muscle representations are more likely encoded in the primary motor cortex (Bernier and Grafton, 2010; Cisek et al., 2003; Kakei et al., 2001; Wiestler et al., 2014). Therefore, in YA visuomotor constraints may interfere with a broader sensorimotor network than load constraints, leading to a stronger inter-limb coupling and a higher level of difficulty.

However, OA in the present study did not show a difference between visuomotor and force constraints (Figure 2A). Specifically, OA scored 40.4 ± 20.2% and 39.37 ± 21.3% in asymmetric contexts with visuomotor and force constraints, respectively. In contrast, YA scored 52.7 ± 7.5% and 59.2 ± 9.1% in these contexts, respectively. On average, OA performance was approximately 12% worse than YA with visuomotor constraints, but 20% worse with force constraints. This suggests that aging has a greater negative effect on overcoming force asymmetry than spatial asymmetry in bimanual coordination. Nevertheless, future studies are needed to investigate the neural mechanisms underlying this difference.

### 4.2 Rightward bias in overt attention during bimanual coordination in YA, but not in OA

The relationship between attention and action has been extensively examined in YA (Baldauf and Deubel, 2010). In the present study, we focused on the overt attention by measuring gaze distribution, i.e., fixation frequency and duration asymmetries. Note that the covert attention cannot be directly measured in this study and our results need to be interpreted with caution. The effect of handedness on overt visual attention during unimanual motor tasks could be task-dependent. Some studies showed that gaze patterns do not differ between right and left hands. For instance, the temporal patterns of gaze during sequential pointing movements and spatial patterns of gaze before reaching to grasp movements are similar between left and right hands (Brouwer et al., 2009; Wilmut et al., 2006). In contrast, other studies demonstrated asymmetrical gaze patterns between two hands. For instance, more corrective saccades are associated with the left hand than the right hand performing in a reciprocal reaching task (Helsen et al., 1998). Furthermore, Rand and Ringenbach (2022) indicated quicker gaze fixation on reaching targets for the non-dominant hand. It was argued that such differences in gaze patterns may be explained by stronger reliance of the right hand on visual feedback to perform predictive control, whereas the left hand may be better at using proprioceptive feedback (Goble and Brown, 2008; Sainburg, 2014).

In bimanual studies, eye movement has often been used as a portal to assess how overt attention is allocated between two limbs. A rightward bias of overt attention has been revealed in a variety of tasks in YA. For example, it was found that gaze tends to be directed to the right target during bimanual reaching to targets of the same difficulty (Honda, 1982; Riek et al., 2003; Sardar et al., 2023). Similarly, it was shown that there is a tendency for participants to fixate on the right hand’s landing positions during a high-speed bimanual cup stacking task (Foerster et al., 2011). These results are consistent with our findings in the symmetric Baseline condition where the fixation was directed toward the right side more frequently and the right-side fixations were longer. Such rightward bias can be explained by the aforementioned advantage of the right hand in using visual feedback, which may result in the superior tracking performance of the right hand in our symmetric condition. However, our results in asymmetric conditions deviated from previous findings that were based on bimanual fast-reaching paradigms. When participants must simultaneously reach to targets of different difficulties with two hands, the gaze tends to shift toward the more difficult side (Riek et al., 2003; Sardar et al., 2023). In contrast, despite subtle changes in fixation frequency and duration, we found that the rightward gaze bias was relatively invariant in conditions where asymmetrical task difficulties were induced by visuomotor and load constraints (Figures 3A, B). Interestingly, we also found that the asymmetric distribution of gaze did not change across the process of adaptation as the performance improved from early to late stages with left-side constraints. Therefore, our results provide strong evidence that the rightward bias in overt visual attention was a robust oculomotor phenomenon in right-handers during continuous bimanual tasks. Even if one can temporarily modulate the visual attention to favor the left side in short-duration fast-reaching tasks, it seems that the rightward bias remains strong in continuous tasks with long duration.

In this research study, we did not find a rightward bias in the overt visual attention in OA, who exhibited symmetric gaze distributions in both symmetric and asymmetric task contexts (Figures 3C, D). Literature about visual attention of OA in motor tasks remains scarce. Nevertheless, there has been extensive evidence that suggests an effect of aging in orienting visual attention in perceptual tasks (Erel and Levy, 2016). An interesting age-related difference has been shown in the phenomenon of pseudoneglect, which refers to a systematic perceptual or visual judgment bias favoring a direction of the visual field. In YA, it was found that pseudoneglect has a leftward bias, which was often attributed to the right hemisphere dominance for visuospatial attention processing (Benwell et al., 2014; Cai et al., 2013; Cavézian et al., 2012; Jewell and Mccourt, 2000). In contrast, such leftward bias is diminished and sometimes reversed in OA (Learmonth and Papadatou-Pastou, 2022). Additionally, a recent study examined the gaze of over 4,000 individuals during free viewing of an image and found that age modulated the directional bias of the first several fixations after the image presentation (Strauch et al., 2024). Our findings of age-related reduction in visual attention asymmetry are consistent with these studies. However, it is important to note that the visual attention in YA favored the right side in our study, instead of the leftward bias found in pseudoneglect. This is likely because our task is a visuomotor bimanual task that is driven by a left-hemisphere network (Serrien et al., 2012; Walsh et al., 2008).

### 4.3 Dominant and non-dominant limbs have asymmetrical adaptation capability in bimanual coordination in YA, but not in OA

Our YA results showed that the left hand did not adapt to the asymmetric contexts regardless of the side to which the constraints were applied. In contrast, the performance of the right hand improved significantly over consecutive trials with constraints applied to the left side, but not the right side (Figure 4). The tracking task we used in this study is a type of bimanual coordination that requires independent control of each limb, therefore it is possible that the asymmetrical adaptation was partially caused by differences in sensorimotor control between two limbs. Studies using unimanual tasks in YA revealed that the rates of adaptation to visuomotor rotation and force field for two limbs were similar (Kirby et al., 2019; Schabowsky et al., 2007). However, numerous studies have found that the underlying adaptation mechanisms may be different between two limbs, which led to asymmetrical aftereffects (Duff and Sainburg, 2007), asymmetrical inter-limb transfer (Criscimagna-Hemminger et al., 2003; Sainburg and Wang, 2002), different movement characteristics (Yadav and Sainburg, 2014), or different cortical activities (Kirby et al., 2019). Such differences were often interpreted within the dynamic dominance framework in which the non-dominant (right) hemisphere is specialized in impedance control whereas the dominant (left) hemisphere is specialized in predictive trajectory control (Sainburg, 2016). Our tracking task can be categorized as trajectory control because there was no unpredictable external perturbation. Therefore, the adaptation in the right hand of YA in this study can be explained by the superior ability of the dominant hemisphere in predictive control. In contrast, the lack of adaptation in the left hand may be explained by the lack of demand for impedance control which is more adept at stabilizing against environmental uncertainty.

However, the interlimb difference in unimanual control mechanisms cannot explain our results about the right hand showing no adaptation when the constraints were added to the right side. We speculate that this finding can be attributed to the interlimb coupling effects. Previous studies have shown that the interlimb coupling effects can be asymmetrical in response to different task demands. For example, the inter-limb interference was stronger when the left hand must produce various levels of force while the right hand maintained a constant force amplitude, than when the roles of the hands were reversed (Hu and Newell, 2011, 2012; Kennedy et al., 2017). Moreover, adaptation of the visible right hand to visuomotor rotation in bimanual reaching caused the invisible left hand to deviate from normal visuomotor mapping. Such interlimb interference was less when the roles of the two hands were reversed (Kagerer, 2015). These findings suggest that the non-dominant hemisphere may be more strongly influenced by the dominant hemisphere than vice versa during bimanual control in YA. This proposition has been supported by several neural imaging studies in which stronger activations of the dominant hemisphere and stronger dominant to non-dominant hemisphere connections were often observed during rhythmic bimanual tasks (Gross et al., 2005; Maki et al., 2008; Pollok et al., 2005; Serrien et al., 2003; Viviani et al., 1998). In our study, the adaptation can be considered as a process to overcome the inter-limb coupling effect that caused the performance impairment in asymmetric contexts. We speculate that the demand for the dominant hemisphere to maintain independent control (i.e., reducing inter-limb coupling) interfered with the ability of the dominant hemisphere to adapt to the right-side constraints. In contrast, such interference was weaker in conditions with left-side constraints, which have a lower demand for maintaining independent control.

In contrast to YA, OA demonstrated a diminished capacity for differential adaptation between conditions with left- and right-side constraints (Figure 4). One explanation is that OA’s symmetrical overt attention (Figure 3) did not benefit either side, whereas YA’s rightward bias in overt attention may facilitate the adaptation of the right hand in conditions with left-side constraints. Additionally, aging may have asymmetrical effects on the visuomotor control of dominant and non-dominant limbs. It has been shown that aging is associated with a more pronounced decline in the right hand’s performance in unimanual tasks. For instance, the superiority of the dominant hand in tracking and fast-reaching precision was only found in YA but not in OA (Kalisch et al., 2006; Przybyla et al., 2011). Moreover, a reduction of asymmetry in the interlimb transfer of unimanual visuomotor adaptation was found in OA (Wang et al., 2011). Therefore, it is possible that the lack of right-hand adaptation in OA in our study was caused by a weakened ability of the dominant hand to adapt to the constraints. Alternatively, our findings can be explained by age-related decline in the ability to inhibit interhemispheric crosstalk (Maes et al., 2017). For bimanual tasks, it has been shown that OA often had poorer performance when two limbs were required to produce complex asymmetrical patterns (Fling and Seidler, 2012; Kiyama et al., 2014; Spirduso and Choi, 1993; Summers et al., 2010), indicating a weakened ability to control two limbs independently. It was suggested that the interhemispheric inhibition via corpus callosum is critical to independent processing of both hemispheres (Gooijers and Swinnen, 2014), and that age-related changes in callosal size and integrity play a critical role in bimanual control deficits (Fling et al., 2011).

### 4.4 De-differentiation of visuomotor control in OA

Our results demonstrated a lack of asymmetry in OA. Both overt visual attention and motor behavior did not differ between the right and left sides. One explanation for these results is that the OA may consider their left hand to be inferior and learned to use a compensatory strategy that pay more visual attention to the left hand during bimanual tasks. Despite the changes of limb dominance measured as motor performance and limb use in daily activities, OA are not less right-handed than YA using self-rated measures (Kalisch et al., 2006; Papadatou-Pastou et al., 2020). Therefore, it is possible that the OA developed a more balanced attention distribution across life span for their perceived handedness and they think that this is an appropriate strategy in this experimental task. Alternatively, the visuomotor control of OA may have adapted to the age-related changes of each limb as well as changes in the interhemispheric coupling. As the superiority of the dominant limb performance decreases and the interlimb coupling increases with age (see above), shifting the attention distribution to be more equal between two limbs may represent a more optimal motor control.

Our findings are consistent with a broad literature showing an age-related de-differentiation process in the brain (Hill et al., 2020). For instance, many neuroimaging and neural stimulation studies revealed less asymmetric activation patterns across brain areas in OA than in YA during various unimanual motor tasks (Carp et al., 2011; Dolcos et al., 2002; Lord and Ward, 1994; Matsunaga et al., 1998; Mattay et al., 2002; Naccarato et al., 2006; Riecker et al., 2006), and bimanual motor tasks (Coxon et al., 2010; Goble and Brown, 2008). Researchers have proposed several theoretical frameworks to explain such phenomena. Hemispheric Asymmetry Reduction in Older Adults (HAROLD) model suggests that as the brain ages, it compensates for declines in neural efficiency by engaging both hemispheres more bilaterally (Cabeza, 2002). Alternatively, the Right-Hemi Aging Model suggests that the right hemisphere experiences a faster decline than the left hemisphere, which could affect spatial and visuomotor functions (Weller and Latimer-Sayer, 1985). Both of these models may lead to decreased functional specificity of the two hemispheres, which align with the visuomotor symmetry in OA observed in our results. Furthermore, other age-related changes in how the visuomotor control uses neural resources, such as subcortico-cortical activation shift (Maes et al., 2017) and posterior-anterior shift (Davis et al., 2008), may also play a role in our results. Future imaging studies are needed to determine the cortical mechanisms underlying eye-hand coordination in OA.

### 4.5 Limitations

The main limitation of the proposed study is the small sample size. First, our sample size was estimated based on an *a priori* power analysis that assumed a Cohen’s f effect size of 0.3, beta 0.8, and alpha 0.05 in a 3-way repeated measures ANOVA design within each age group. However, the current sample size cannot detect small differences in a mixed design with Age as a factor. This limited direct statistical comparison between age groups for the asymmetric conditions. Second, we only included right-handed participants. Left-handers are not mirror image of right handers, and their visuomotor behavior in relation to handedness can be very different from right handers (Buckingham and Carey, 2014). Therefore, the present study cannot be generalized to left handers. Lastly, our participant pool consisted primarily of younger adults and OA over the age of 55, with no representation from individuals above 75 years old. Given that age-related declines in motor and cognitive function tend to accelerate in this older-old age range (Pothier et al., 2015), it is possible that the patterns of adaptation and coordination observed in the proposed study would differ significantly. Despite these limitations, we believe our results using a small sample size still revealed insightful findings about the differences between YA and OA. Future studies with a much larger sample size are needed to fully characterize the effects of aging on the visuomotor control of bimanual coordination in a broader population.

## 5 Conclusion

This research demonstrates key age-related differences in bimanual coordination under asymmetric task constraints, revealing both motor control and visual attention distinctions between two age groups. YA encountered more difficulty with visuomotor asymmetry compared to force asymmetry, showing higher performance errors when each limb faced different spatial demands. In contrast, OA displayed similar levels of difficulty across both visuomotor and force asymmetries, confirming that aging affects the ability to adapt to varying constraint types. Moreover, YA exhibited a distinct rightward bias in visual attention during tasks under either symmetric or asymmetric task demands. However, OA showed no such attentional bias, instead maintaining a symmetrical gaze distribution across screen for different contexts. This lack of lateralized attention may reflect age-related changes in hemispheric specialization. Furthermore, YA demonstrated asymmetrical adaptation capabilities, with the dominant right hand showing significant performance improvement when the left side was constrained. Conversely, OA lacked this differential adaptation. This age-related de-differentiation in visuomotor control, observed both in motor behavior and gaze allocation, highlights a general decline in lateralized control mechanisms with age. Overall, our hypotheses were supported by our findings. Future research is needed to further clarify these mechanisms and to develop targeted rehabilitation protocols that address these age-related visuomotor changes, optimizing functional outcomes for OA in tasks requiring bimanual coordination.

## Data Availability

The raw data supporting the conclusions of this article will be made available by the authors, without undue reservation.
